# Tracking Transplanted Stem Cells Using Magnetic Resonance Imaging and the Nanoparticle Labeling Method in Urology

**DOI:** 10.1155/2015/231805

**Published:** 2015-08-27

**Authors:** Jae Heon Kim, Hong J. Lee, Yun Seob Song

**Affiliations:** ^1^Department of Urology, Soonchunhyang University Hospital, College of Medicine, Soonchunhyang University, Seoul, Republic of Korea; ^2^Biomedical Research Institute, Chung-Ang School of Medicine, Seoul, Republic of Korea

## Abstract

A reliable *in vivo* imaging method to localize transplanted cells and monitor their viability would enable a systematic investigation of cell therapy. Most stem cell transplantation studies have used immunohistological staining, which does not provide information about the migration of transplanted cells *in vivo* in the same host. Molecular imaging visualizes targeted cells in a living host, which enables determining the biological processes occurring in transplanted stem cells. Molecular imaging with labeled nanoparticles provides the opportunity to monitor transplanted cells noninvasively without sacrifice and to repeatedly evaluate them. Among several molecular imaging techniques, magnetic resonance imaging (MRI) provides high resolution and sensitivity of transplanted cells. MRI is a powerful noninvasive imaging modality with excellent image resolution for studying cellular dynamics. Several types of nanoparticles including superparamagnetic iron oxide nanoparticles and magnetic nanoparticles have been used to magnetically label stem cells and monitor viability by MRI in the urologic field. This review focuses on the current role and limitations of MRI with labeled nanoparticles for tracking transplanted stem cells in urology.

## 1. Introduction

Molecular imaging technologies have evolved recently and facilitate functional monitoring and evaluation of genes and organs for their roles in health and disease [1, 2]. Stem cell transplantation has good prospects for clinical application. However, the challenges in molecular imaging are to develop effective imaging strategies with a combination of imaging modalities, labeling reporter systems, and probes. Several studies have used magnetic resonance imaging (MRI) to trace transplanted stem cells in animal models [3, 4].

Several molecular imaging modalities including positron emission tomography (PET), MRI, and newer modalities are based on transmitting light through tissues, such as* in vivo* bioluminescence imaging and fluorescence imaging. Among them, MRI is the most popular imaging modality. MRI used in conjunction with magnetically labeling is a powerful technique for noninvasively detecting and tracking transplanted cells in longitudinal animal studies [1, 2, 5].

Labeling materials have great importance in the field of molecular imaging. Labeling stem cells makes merged cells distinguishable from host cells to follow transplanted stem cells.

Molecular materials for labeling should first reveal cellular and molecular processes throughout the entire study period. Secondly, the probes should be highly sensitive to small changes in cell function and distribution. Finally, they should not significantly alter the labeled biological process itself [1, 2, 5].

Gadolinium and ferric oxide are two common cell labeling contrast media used during MRI [6]. New technologies with tumor targeting and drug delivery are being conceptualized. Developments in nanotechnology have provided more innovative and effective approaches in various areas of clinical research, such as diagnosis [7], monitoring [8, 9], and therapy [10–12]. Labeling with nanoparticles is an emerging trend, particularly in oncology such as “cancer nanotheranostics,” which includes simultaneous imaging and treating cancer cells by applying nanoparticles [13].

Although many studies have investigated the efficiency of molecular imaging using MRI with labeled nanoparticles, few studies are available in the urologic field. The aim of this study was to review MRI and labeling techniques for tracking transplanted stem cells (physiological labeling) in the urologic field and to review the characteristics and limitations of current nanoparticle labeling methods.

## 2. Stem Cell Labeling

Cell labeling can be divided into physical cell labeling and reporter gene imaging. Physical cell labeling is completed before cell administration and can be accomplished with superparamagnetic iron oxide (SPIO) particles for MRI [14, 15] and radionuclide labeling for single-photon emission computed tomography [16] and PET [17]. In reporter gene imaging, a gene coding synthesis of a detectable protein is introduced into a target cell line or tissue via viral or nonviral vectors. As a result, changes in signals following cell administration can be used as indicators of cell proliferation and death [18].

Many labeling techniques involve incubating cells and use of transfection agents. The different magnetic labeling techniques result in a considerable increase in the cellular iron content [26], which is 100 times greater than physiological levels [27]. The largest amount of intracellular iron oxide particles and the use of high-resolution gradient echo sequences allow for highly sensitive* in vivo* MRI methods for detecting viability and efficiency of transplanted stem cells.

**Figure 1:**
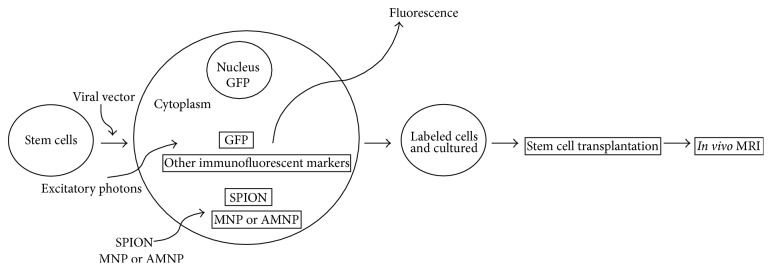
Schematic imaging of stem cell tracking using magnetic resonance imaging (MRI) combined with nanoparticle labeling.

Most studies have tracked physically labeled transplanted stem cells (Figure 1). Migration of lymphocytes [28], hematopoietic stem cells [29], mesenchymal stromal cells (MSCs) [30], neuronal precursor cells [31], and tumor cells [32] has been demonstrated in different disease models using* in vivo* MRI. Specific cancer stem cell antigens or receptors have been detected by reporter gene imaging.

Application of conventional GFP-like fluorescent proteins, including eGFP, DsRed, and mCherry, has limitations due to the penetration depths of visible light in the body [33]. To overcome this limitation, near-infrared fluorescent protein (IFP) has been developed from the DrBphP bacterial phytochrome of* Deinococcus radiodurans* and showed the possibility in the application of IFPs for protein labeling and* in vivo* tracking imaging [5].

## 3. Iron Oxide Nanoparticles

The original iron oxide nanoparticles were developed in 1995 and were Dextran-coated iron oxide nanoparticle with a 100–150 nm hydrodynamic radius and contained a 5–10 nm iron oxide core [34]. These standard, well-characterized iron oxide nanoparticles have been used widely, but low labeling amounts and efficiencies were shortcomings [35]. To overcome this weak point, simple transfection agents were combined with ferumoxides enabling robust labeling of a number of cell types [36].

Most recently, a nanomaterial consisting of a mixture of ferumoxytol, heparin sulfate, and protamine sulfate has been reported and can be used to safely label various types of cells for tracking by MRI [8]. Ferumoxytol, heparin sulfate, and a protamine sulfate conjugate is currently the most popular material for physical cell labeling in urology [37]. Dextran-coated superparamagnetic iron oxide nanoparticles (SPIONs) and micron-sized iron oxide particles were developed for MRI-based cell tracking [26, 38]. The size of iron oxide particles for cell labeling ranges from very small particles to micron-sized particles, and SPIO is a medium-sized particle [39–41].

Among the different types of nanoparticles, SPIONs are promising candidates for use with molecular imaging modalities due to their superparamagnetic behavior and surface-modification properties. One of the important features of SPIONs is that they lose their magnetism and become highly dispersed when the magnetic field is switched off, which prevents easy recognition and engulfment by macrophages [42].

As SPIONs are biodegradable and biocompatible, they can be applied in various biomedical fields, such as magnetofection [43], gene therapy [44], and cell and biological material separation [45]. SPIONs are mostly magnetite (Fe_3_O_4_), and they convert to maghemite when exposed to oxygen. They can be metabolized easily and transported by proteins, such as ferritin, transferrin, and hemosiderin, and they can be stored in endogenous iron reserves of the body for later use [1, 2].

The advantage of applying a magnetic field to guide nanoparticles to their target is to reduce stem cell waste, lower the frequency of stem cell administration, and avoid unwanted side effects [1, 2]. SPIONs are very promising materials for biomedical applications due to their increased ability to covalently attach to various receptors, peptides, antibodies, or ligands [46]. Furthermore, SPIO particles have no adverse effects on viability or proliferation of labeled cells [40, 47].

## 4. Non-SPION Nanoparticles

Most magnetic labeling procedures depend on Dextran-coated magnetic nanoparticles. Several magnetic cell labeling methods have been developed but the most commonly used one is coincubating Dextran-coated nanoparticles with a transfection agent [48].

However, these materials follow a low-efficiency fluid-phase endocytosis pathway and require long incubation times or the use of transfection agents to achieve substantial iron uptake. Moreover, the complexes formed by the nanoparticles and transfection agents are not easily controlled.

Anionic magnetic nanoparticles (AMNPs) have negative surface charges, are free of a Dextran coating, adsorb readily to cell membranes, and are internalized without the need for transfection agents or long incubation times [20, 49, 50]. AMNPs permit controlled uptake by various cell types [20, 51–53]. AMNPs have advantages of easy and rapid absorption and subsequent internalization by endocytosis [50, 54, 55]. AMNP biocompatibility has been demonstrated in many preclinical studies, including local cell grafts for tissue regeneration [20, 56] and immune cell trafficking after systemic injection [52, 57, 58].

Fluorescent magnetic nanoparticles (MNPs) contain rhodamine B isothiocyanate within a silica shell to overcome the negative surface charge. This tagging material does not require a transfection agent during cell labeling, the MNP core is composed of ferrite, and the inner silica shell portion contains fluorescent materials [59]. It has both magnetic and optical features, and Prussian blue staining is not necessary to detect viability and efficiency of transplanted stem cells within tissue.* In vivo* tracking of transplanted MSCs labeled with fluorescent MNPs in a liver cirrhosis rat model by MRI has been reported [60].

Among the six studies that used MRI and physical labeling, three used SPIONs, two used MNPs, and one used AMNPs (Table 1).

Gadolinium (Gd) based contrast agents are normally used to reduce *T*1 period and to give positive contrast in MR images. Available agents are different kinds of gadolinium ion based chelates which are relatively stable molecules. To date, Gd based contrast agent has evolved to be more efficient contrast agents including Gd3+ based agents with higher molecular weight like Gd-DTPA functionalized polymers, Gd-DTPA terminated dendrimers, and Gd complex loaded liposomes as well as high density lipoprotein nanoparticles or micelles [61]. Recently, several new nanoparticles are introduced for* in vivo* imaging including Fe_3_O_4_@SiO_2_ nanoparticles and Gd-DOTA-peptides [62–64].

## 5. Ideal Features of Labeled Nanoparticles

Nanoparticles for any biological application must be biocompatible, nontoxic, and stable at physiological pH. The ideal features are high magnetization and a narrow size distribution. The nanoparticles should have contrast enhancement properties for imaging and tracking of malignant cells/tissues, and their surfaces should be coated with biodegradable material. They should have the ability to conjugate with a range of receptors with high targeting and drug-delivery efficiencies. The half-life should be long, and the zeta-potential should be optimized [1, 5, 9].

## 6. Current Uses for MRI Techniques in Urology

MRI is a widely used powerful imaging technique that provides high resolution in the field of urology. It is used to evaluate stem cells transplanted to urologic organs. MRI alone or MRI with a physical labeling method has been used to monitor the efficiency of cell transplantation, cellular homing, and targeting. MRI has been used in prostate cancer research, bladder dysfunction research, urethral sphincter studies, and a penis study (Table 1).

### 6.1. Prostate Cancer

Molecular imaging combined with a labeling technique has been used to detect specific prostate cancer antigens. Two studies showed the efficiency of MRI for detecting transplanted stem cells as a vector for prodrug therapy. Abrate et al. [21] reported that MRI can be used to follow orthotopic tumor progression. Although those authors did not apply a physical labeling method, they demonstrated that intravenous injections of CD-MSC cells, followed by intraperitoneal administration of 5-fluorocytosine, caused tumor regression in transgenic adenocarcinoma of the mouse prostate model, which develops aggressive and spontaneous prostate cancer.

**Figure 2:**
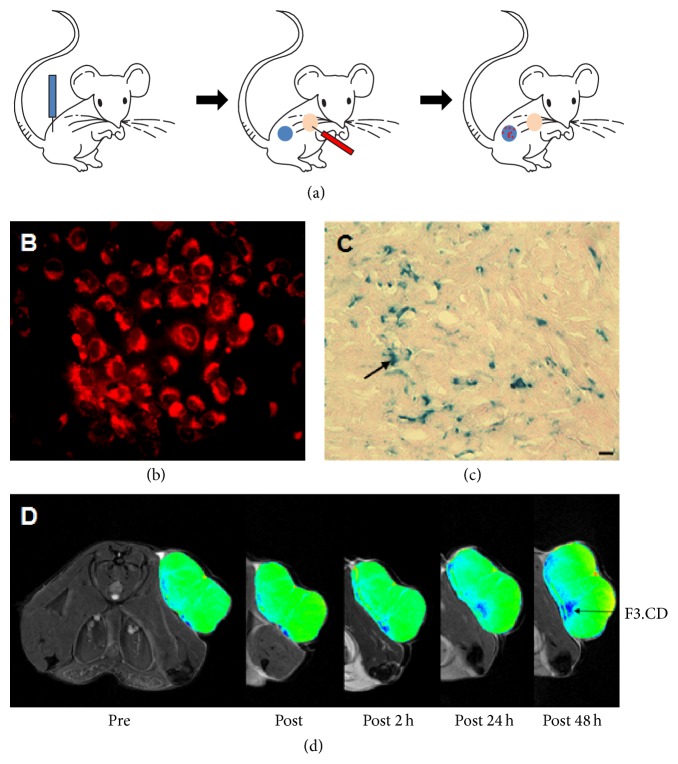
Intravascular delivery of stem cells (HB1.F3.CD) targeting prostate cancer. (a) Schematic illustration of inducing prostate cancer using TRAMPC2, a systemic injection of HB1.F3.CD cells, and migration of the gene-modified stem cells toward the prostate cancer. Blue = TRAMPC2; red = HB1.F3.CD cells. (b) X-gal staining of induced prostate cancer 2 days after injecting HB1.F3.CD cells. Arrow indicates the cells. (c) Magnetic resonance imaging (MRI) of prostate cancer 48 hr after injecting HB1.F3.CD cells into mice. Arrow indicates the cells (scale bar, 100 *μ*m). HB1.F3, neural stem cells; CD, cytosine deaminase.

Lee et al. [19] reported monitoring the migration of genetically modified stem cells by MRI after labeling the cells with fluorescent MNPs. Human neural stem cells encoding CD (HB1.F3.CD) were prepared and labeled with MNPs (Figure 2). HB1.F3.CD stem cells systemically transplanted into tumor-bearing C57B mice migrated toward the tumor, and tumor implant volume decreased significantly in combination with the prodrug 5-FC.

### 6.2. Bladder

Traditionally, many studies showed the efficacy of stem cell treatment in bladder dysfunction [65–67]; however only several studies introduced molecular imaging techniques. Yun and Ja [24] showed similar viability of MSCs loaded with SPIONs compared to unlabeled cells. SPIO-labeled MSCs underwent normal chondrogenic, adipogenic, and osteogenic differentiation. MRI signal intensity in the areas of SPION-labeled MSCs in rat and rabbit bladders decreased and was confined locally. MRI demonstrated that SPION-labeled MSCs injected into the bladder could be seen for at least 12 weeks.

Lee et al. [22] reported that MRI images were useful to track transplanted MSCs in bladder outlet obstruction induced bladder dysfunction. Serial T2-weighted MRI images were taken immediately after transplant of SPION-labeled MSCs and at 4 weeks after transplantation. T2-weighted MRI showed a clear hypointense signal induced by the SPION-labeled MSCs. Collagen and transforming growth factor-*β* expression protein increased after bladder outlet obstruction, and the expression of both returned to original levels after MSC transplantation.

Lee et al. [25] reported the efficiency of MRI for tracking transplanted MSCs in a spinal cord injury-induced bladder dysfunction model. MNP-labeled B10 cells were injected into the bladder wall 4 weeks after the spinal cord injury. Serial MRI was taken immediately after MNP-B10 injection and at 4 weeks after transplantation.

### 6.3. Penis

Song et al. [23] suggested that MRI can be used to investigate the long-term therapeutic potential of MSCs to treat erectile dysfunction. SPION-labeled MSCs injected into the corpus cavernosa of rats and rabbits were evaluated noninvasively by molecular MRI. MRI signal intensity at the areas of SPION-labeled MSCs in the rat and rabbit corpus cavernosa decreased and was confined locally. MRI demonstrated that the MSCs could be observed for at least 12 weeks after injection into the corpus cavernosum.

### 6.4. Urethral Sphincter

Rivière et al. [20] magnetically labeled muscle implants with AMNPs. They evaluated the biocompatibility of the labeling procedure and its utility for noninvasive MRI follow-up of cell therapy in a female pig model. AMNPs were adsorbed onto the implant surface of myogenic precursor cells and were magnetically labeled within the implants. Magnetic labeling did not affect cell proliferation or differentiation. Autograft detection* in vivo* by 0.3-T MRI was possible for up to 1 month.

## 7. Discussion

Although molecular imaging techniques have evolved significantly during the last decade, no single imaging modality can provide all the information required to track transplanted stem cells and monitor their functional effects. Each imaging modality used for stem cell tracing has its advantages and disadvantages [54, 68]. PET has high sensitivity for tracking biomarkers* in vivo* but lacks the ability to provide detailed anatomic structure. Optical imaging has high molecular sensitivity but provides less anatomical localization and is mainly used in small animals. MRI coupled with physical labeling has high resolution and the capabilities of three-dimensional anatomical imaging. However, MRI has low sensitivity for cell tracing. Moreover, it cannot detect cell number or location by cell division because cell division may dilute intracellular markers with the shedding of iron particles [69]. Hence, it is necessary to combine complementary imaging methodologies as with multimodality imaging approaches.

Some nonspecific signal problems occur with different imaging modalities as a result of dead transplanted cells. For example, dead transplanted cells containing iron oxide nanoparticles may result in MRI signals representing macrophage phagocytosis of labeled cell debris [1, 2, 5]. Limitations continue in the basic knowledge about the pivotal biological characters of transplanted stem cells, such as survival, integration, and migration, and the influence of the host microenvironment. Despite the potential for biomedical applications, SPIONs face some targeting and imaging limitations. The proportion of SPIONs that reach the target through surface-bound antibodies is low, thereby limiting their application for imaging and drug delivery.

MRI is a commonly used imaging modality and it could be used in a large animal model. To detect successful delivery and subsequent migration using iron oxide–based agents,* ex vivo* labeling of the stem cells are required [1]. The robust negative contrast image generated by iron oxide agents has shown efficient cell labeling. Studies have also reported successful tracking at near single-cell resolution [70]. However, to assess the viability additional techniques are needed such as using promoter genes, engineering cells to overexpress transferrin receptors, and nongenomic technique using manganese-enhanced MRI.

To date, the important pitfalls of MRI with SPIO labeled cells or luciferase-based bioluminescent imaging are that these modalities could provide the information about cell survival, anatomical coregistration of engrafted cells together with real time, and image-guided delivery. To overcome these limitations, chemical exchange saturation transfer is an emerging MRI contrast mechanism based on the use of radiofrequency saturation pulses to detect agents containing protons that exchange rapidly with water [27]. Chan et al. [9] reported that pH nanosensor-based MRI technique can monitor cell death* in vivo* noninvasively. They demonstrated that specific MRI parameters related with cell death of microencapsulated hepatocytes are associated with the measured bioluminescence imaging radiance.

To overcome this problem, classic physical labeling must be upgraded with a reporter gene imaging technique. In fact, this combined modality is being investigated in cancer stem cell studies. SPIONs with specific tumor-targeting ligands and sensitive imaging probes must be developed [71]. To date, most* in vivo* imaging studies showed the limitations in detecting the engraftment of stem cells [72].

Another major problem facing drug delivery using nanoparticles is the burst effect. When drug-coated nanoparticles are injected into a system, a significant quantity of the drug is liberated suddenly due to alterations in the physiological host environment, which can be dangerous to the patient [1, 2, 5]. To overcome this effect, nanoparticles must be cross-linked with polymers or incorporated into a polymer matrix that provides more rigidity and helps provide sustained drug delivery under* in vivo* conditions for longer times [1, 2, 5]. Mahmoudi and Laurent [73] demonstrated increased stability of drug-loaded SPIONs under* in vivo* conditions using a PEG-cofumarate cross linking agent. Other issues that must be solved regarding nanoparticles include toxicity, gene alternations, penetration of the blood-brain barrier, and colloidal stability [1, 2, 5].

Molecular imaging combined with a nanoparticle labeling method is useful not only for physical labeling to monitor stem cells but also to detect prostate cancer antigens. Recent preclinical studies on multimodal molecular imaging methods have the potential to be helpful for noninvasive prostate cancer diagnosis and image-guided immunotherapy [74, 75]. Multiple groups are actively pursuing the development of imaging probes for cellular and molecular MRI [75–79]. More studies are needed to develop various molecular markers, including ligands, antibodies, and peptides that can easily bind MRO probes.

*In vivo* imaging represents a dedicated platform to evaluate and quantify molecular and cellular events related to cellular engraftment. This integrative approach should be more developed with validation for systematic translation of stem cell therapy.

## 8. Conclusions

Molecular imaging is a new discipline that allows for* in vivo* cellular and molecular imaging of pathophysiological processes and the results of therapeutic interventions. MRI is a contending and complementing modality for stem cell studies in urology. MRI can be used to evaluate migration and survival of transplanted stem cells in prostate cancer and bladder dysfunction models. It has also shown potential utility for use on erectile dysfunction and urethral sphincter dysfunction. Noninvasive imaging methods using MRI have the advantage of longitudinal monitoring of transplanted stem cells in animals.

## Figures and Tables

**Table 1:** Studies using stem cell-based gene therapy for prostate cancer.

Study	Stem cell	Animal	Organ	Nanoparticle	Labeling viability	Labeling efficiency	Iron quantification	*In vivo* MRI	*In vitro* MRI	Labeling dose
Lee et al. [19]	Human NSC	C57BL/6 mice	Metastatic prostate cancer	MNP	Immunofluorescence microscope, X-gal staining	None	None	T2-weighted gradient-echo	None	100 *μ*g/mL MNP

Rivière et al. [20]	Pig MPC	Pig	Urethral sphincter	AMNP	Indirect antidesmin immunofluorescence	Prussian blue staining and electron microscopic imaging	Magnetophoresis, electron spin resonance	T1-weighted gradient-echo	T1, T2-weighted gradient-echo	4.5 × 10^9^ AMNPs

Abrate et al. [21]	Human MSC	C57BL/6 mice	Metastatic prostate cancer	None	None	Prussian blue staining and electron microscopic imaging	None	T2-weighted RARE images	None	None

Lee et al. [22]	Human MSC	Rat	Bladder	SPION	Trypan blue staining	Prussian blue staining	None	T1-weighted gradient-echo	None	25 *μ*g/mL SPION

Song et al. [23]	Human MSC	Rat, rabbit	Penis	SPION	Trypan blue staining	Prussian blue staining and electron microscopic imaging	Nome	T2-weighted gradient-echo	T1-weighted gradient-echo	25 *μ*g/mL SPION

Yun and Ja [24]	Human MSC	Rat, rabbit	Bladder	SPION	Trypan blue staining	Prussian blue staining and electron microscopic imaging	None	T2-weighted gradient-echo	T2-weighted gradient-echo	25 *μ*g/mL SPION

Lee et al. [25]	Human NSC	Rat	Bladder	MNP	Immunofluorescence microscope	None	None	T2-weighted gradient-echo	None	100 *μ*g/mL MNP

NSC: neural stem cell; MNP: magnetic nanoparticle; MPC: myogenic precursor cell; MSC: mesenchymal stem cell; SPION: super paramagnetic iron oxide nanoparticle.
